# TROP-2 overexpression in papillary renal cell carcinoma supports its potential as a therapeutic target for antibody-drug-conjugate therapy

**DOI:** 10.1007/s00345-025-05880-2

**Published:** 2025-09-01

**Authors:** Carolina Kessler, Melanie von Brandenstein, Niklas Klümper, Philipp Krausewitz, Enno Storz, Constantin Rieger, Laurenz Sperber, Pia Paffenholz, Yuri Tolkach, Ralph Wirtz, Markus Eckstein, Axel Heidenreich, Richard Weiten

**Affiliations:** 1 Department of Urology, Uro-Oncology, Robot-Assisted and Specialized Urologic Surgery, University Hospital Cologne, Kerpener Str. 62, 50937 Cologne, Germany; 2 Department of Urology, University Hospital Bonn, Bonn, Germany; 3 Institute of Experimental Oncology, University Hospital Bonn, Bonn, Germany; 4 Institute of Pathology, University Hospital Cologne, Cologne, Germany; 5 STRATIFYER Molecular Pathology GmbH, Cologne, Germany; 6 Institute of Pathology, University Hospital Erlangen, Friedrich-Alexander-Universität Erlangen-Nürnberg, Erlangen, Germany; 7 Department of Urology, Medical University Vienna, Vienna, Austria

**Keywords:** Antibody-drug conjugates, Renal cell carcinoma, Sacituzumab govitecan, TROP-2

## Abstract

**Objective:**

To evaluate the expression of trophoblast cell surface antigen-2 (TROP-2), a broadly expressed antibody-drug conjugate (ADC) target, in non-clear cell renal cell carcinoma (nccRCC), and to perform a proof-of-concept analysis assessing the cytotoxic efficacy of the TROP-2-directed ADC Sacituzumab govitecan (SG) in RCC cell lines.

**Methods:**

A cohort comprising clear cell RCC (ccRCC, *n* = 44), papillary (pRCC, *n* = 22), chromophobe (chRCC, *n* = 22), and benign renal tumors (*n* = 8, including oncocytoma and angiomyolipoma) was analysed using reverse transcription quantitative PCR (RT-qPCR), immunohistochemistry (IHC) with H-score quantification, and enzyme-linked immunosorbent assay (ELISA). In RCC cell lines, TROP-2 protein levels were assessed by Western blotting and flow cytometry, and SG cytotoxicity was evaluated using MTT assays.

**Results:**

TROP-2 mRNA levels were significantly elevated in pRCC compared to ccRCC, chRCC and benign renal tumors (*p* < 0.001). IHC revealed moderate to strong membranous TROP-2 expression in most pRCC cases [*n* = 20/22 with H-score ≥ 100, median H-score 265 (IQR 202.5–290)], while TROP-2 expression was absent or weak in ccRCC and chRCC (*p* < 0.0001). Soluble TROP-2 was detectable in patient serum of RCC patients and strongly correlated with tissue expression (ρ = 0.78, *p* = 0.0001, R^2^ = 0.52). In vitro, TROP-2-positive Caki-1 cells exhibited significant growth inhibition after SG treatment, whereas TROP-2-negative 769-P cells showed resistance (*p* < 0.01).

**Conclusion:**

The selective overexpression of TROP-2 in pRCC, and its functional relevance demonstrated in vitro, provide compelling preclinical evidence supporting TROP-2 as a therapeutic target. These findings support further investigation of TROP-2-directed ADCs, such as SG, in patients with metastatic TROP-2-positive pRCC.

**Supplementary Information:**

The online version contains supplementary material available at 10.1007/s00345-025-05880-2.

## Introduction

Clear cell renal cell carcinoma (ccRCC) represents the most prevalent subtype of renal cell carcinoma (RCC), accounting for approximately 75–80% of all cases [1–3]. The remaining 20–25% consist of non-clear RCC (nccRCC), which includes papillary RCC (pRCC), chromophobe RCC (chRCC), and other rare histological variants [1–4]. Among these, pRCC is the most frequent nccRCC subtype, comprising approximately 15% of all RCC cases [1–3]. Despite this histopathological heterogeneity the majority of therapeutic advances and clinical trials to date have been centered on ccRCC, resulting in limited evidence-based treatment options for patients with nccRCC [1–5].

The recently published ARON-1 study, a large multicenter, retrospective, real-world analysis involving 200 patients with metastatic pRCC treated across 40 centers in 12 countries, provided valuable insight into current treatment outcomes for this population [3]. The study demonstrated that immune-oncology (IO)-based combinations significantly improved median progression-free survival (PFS) compared to tyrosine kinase inhibitors (TKIs) (17.4 months vs. 6.4 months, *p* < 0.001). IO-based therapies were also associated with superior 1- and 2-year PFS rates, as well as a higher objective response rate (ORR: 41% vs. 27%, *p* = 0.037). Although the difference in median overall survival (OS) between the two groups did not reach statistical significance (28.8 vs. 22.5 months, *p* = 0.081), IO-based regimens yielded significantly improved 1-year and 2-year OS rates (*p* = 0.038 and *p* = 0.023, respectively) [3]. These findings underscore the need for continued exploration of new therapeutic strategies in pRCC, particularly given the historically poor prognosis and absence of robust second-line treatment options in metastatic nccRCC.

Antibody-drug conjugates (ADCs) represent a promising class of targeted therapies that couple the specificity of monoclonal antibodies with the cytotoxic potency of chemotherapeutic agents. By delivering a toxic payload directly to tumor cells via a tumor-associated antigen, ADCs aim to enhance antitumor efficacy while minimizing systemic toxicity [6–8]. Several ADCs have demonstrated clinical efficacy in epithelial malignancies such as breast, lung, and urothelial cancers [7, 8].

Trophoblast cell surface antigen-2 (TROP-2) is a transmembrane glycoprotein that is overexpressed in multiple epithelial tumors and has been implicated in aggressive tumor biology and poor clinical outcomes [9–12]. Sacituzumab govitecan (SG) is a TROP-2-directed ADC composed of a humanized anti–TROP-2 monoclonal antibody (RS7) conjugated to SN-38, the active metabolite of irinotecan, via a moderately stable linker [13]. SG has demonstrated significant clinical activity in triple-negative breast cancer and metastatic urothelial carcinoma, leading to regulatory approvals based on the ASCENT and TROPHY-U-01 trials [11, 14].

In this study, we investigated TROP-2 expression in nccRCC, with a particular focus on pRCC, utilizing reverse transcription quantitative PCR (RT-qPCR), immunohistochemistry (IHC), and enzyme-linked immunosorbent assay (ELISA). Additionally, we evaluated the preclinical antitumor activity of SG in RCC cell lines and assessed serum TROP-2 levels as a potential surrogate marker of tumor expression.

## Materials and methods

### Patient cohort

A retrospective analysis was conducted on a cohort of 88 patients diagnosed with renal cell carcinoma (RCC), comprising clear cell RCC (ccRCC, *n* = 44), papillary RCC (pRCC, *n* = 22), and chromophob RCC (chRCC, *n* = 22). Additionally, tumor specimens from eight patients with benign renal tumors (oncocytoma and angiomyolipoma) were included. All patients underwent partial or radical nephrectomy between 2018 and 2023 at the Department of Urology, University Hospital of Cologne.

A control group of 17 individuals without known oncological disease was included. These individuals, with a median age of 54 years and a male-to-female ratio of 1:1, underwent routine urological procedures (e.g., stone extraction or transurethral resection of the prostate) and had no evidence of malignancy.

Serum samples were collected from RCC patients and controls preoperatively, on the day of or the day preceding surgery, to enable correlation with corresponding tumor tissue.

Clinicopathological characteristics are summarized in Table 1. Histopathological diagnoses were classified in accordance with the 8th edition of the TNM classification of malignant tumors and the 5th edition of the WHO classification of tumors of the urinary system and male genital organs [15].

**Table 1:**
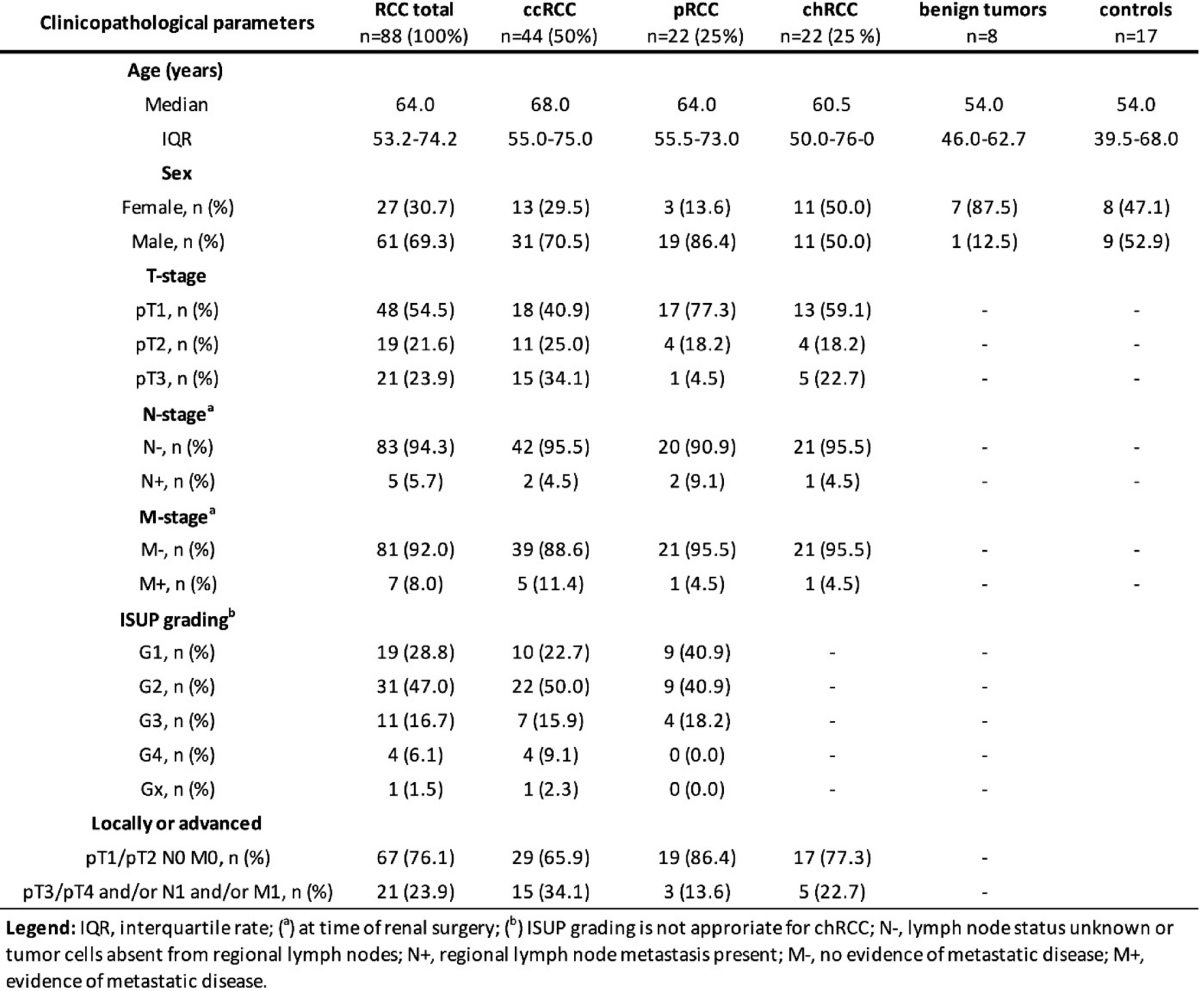
Clinicopathological characteristics for the entire cohort

The study was approved by the Ethics Committee of the Medical Faculty of the University of Cologne (approval number: 23-1178) and conducted in accordance with the Declaration of Helsinki. Written informed consent was obtained from all participants.

### RNA extraction and reverse transcription quantitative PCR (RT-qPCR)

Total RNA was isolated from formalin-fixed, paraffin-embedded (FFPE) tumor specimens using a validated bead-based protocol (STARTIFYER Molecular Pathology GmbH, Cologne, Germany) [16]. TROP-2 mRNA expression was quantified by RT-qPCR using CALM2 as a reference gene, as previously described [16]. PCR conditions included an initial incubation at 50 °C for 5 min, denaturation at 95 °C for 20 s, followed by 40 cycles of 15 s at 95 °C and 60 s at 60 °C.

### Immunohistochemistry (IHC)

Membranous TROP-2 expression was evaluated on FFPE sections using an automated BOND-MAX immunostainer (Leica Biosystems, Wetzlar, Germany). A monoclonal anti–TROP-2 antibody (ab214488, Abcam, UK; 1:1000 dilution) was applied and incubated at 37 °C for 32 min. Staining was independently assessed by two experienced pathologists (Y. Tolkach, M. Eckstein) using the H-score method, with expression classified as weak (H-score 15–99), moderate (100–199), or strong (200–300) [17, 18].

### Enzyme-linked immunosorbent assay (ELISA)

Serum levels of TROP-2 were measured using a semi-quantitative ELISA [19]. A monoclonal anti–TROP-2 antibody (ENZ-ABS380-0100, ENZO Life Sciences, USA; 1:500 dilution) was used, and fluorescence signals were recorded with the FLUOstar Omega^®^ plate reader (BMG Labtech, Germany).

### Cell lines and culture conditions

The RCC cell lines ACHN, A-498, 769-P, 786-O, and Caki-1 were obtained from the American Type Culture Collection (ATCC, USA). ACHN is derived from a pRCC metastasis, while the remaining lines originate from ccRCC. Cells were cultured in RPMI 1640, DMEM, or McCoy’s 5 A medium (PAN-Biotech or Thermo Fisher Scientific, Germany), supplemented with 10% heat-inactivated fetal bovine serum and 0.8% penicillin–streptomycin, and maintained at 37 °C in a humidified 5% CO₂ incubator.

### Western blot analysis

Protein lysates were prepared from cells at 80–90% confluency using RIPA buffer with protease inhibitors. Lysates were separated by SDS-PAGE, transferred to nitrocellulose membranes, and blocked with 5% non-fat milk in TBST. Membranes were incubated overnight at 4 °C with primary antibodies against TROP-2 (ab214488, Abcam, UK; 1:2000) and GAPDH (sc-47724, Santa Cruz Biotechnology, USA; 1:1000), followed by HRP-conjugated secondary antibody (7074, Cell Signaling Technology, USA; 1:2500) for 1 h. Signal detection was performed using the ChemoStar ECL Imager (INTAS, Germany).

### Flow cytometry

Caki-1 and 769-P cells were prepared as single-cell suspensions and stained with anti-human TROP-2 antibody (130-115-098, Miltenyi Biotec, Germany; 1:100) in the presence of Fc block (TrueStain FcX™, BioLegend, USA). Flow cytometry was performed using a BD FACSCanto II cytometer and analyzed with FlowJo software (v10.8, BD Biosciences). A total of 1 × 10⁶ cells were analyzed per sample.

### Cell viability assay

RCC cells were seeded at 1 × 10⁴ cells per well in 48-well plates and treated with increasing concentrations of Sacituzumab govitecan (SG; 0–50 µg/mL) for 72 h. Cell viability was assessed using the MTT assay (ab211091, Abcam, UK), with absorbance measured at 590 nm using the VICTOR Nivo plate reader (PerkinElmer, Germany). Results were normalized to untreated controls.

### Statistical analysis

All statistical analyses were performed using SPSS v28.0.1.1 (IBM Corp.) and GraphPad Prism v9.4.0. Comparisons between two groups were conducted using the Mann-Whitney U test or unpaired t-test, while multiple group comparisons were analyzed using the Kruskal-Wallis test. Receiver operating characteristic (ROC) curve analysis was used to assess the diagnostic performance of serum TROP-2, including area under the curve (AUC), 95% confidence intervals (CI), and p-values. Associations between TROP-2 expression and clinicopathological parameters were tested using Fisher’s exact test or Chi-squared test. Linear regression models were applied to calculate explained variance (adjusted R²). Effect sizes, correlation coefficients, and regression outputs were interpreted in accordance with Cohen’s guidelines [20]. Two-sided p-values < 0.05 were considered statistically significant.

## Results

### Increased TROP-2 mRNA expression in papillary RCC

**Fig. 1:**
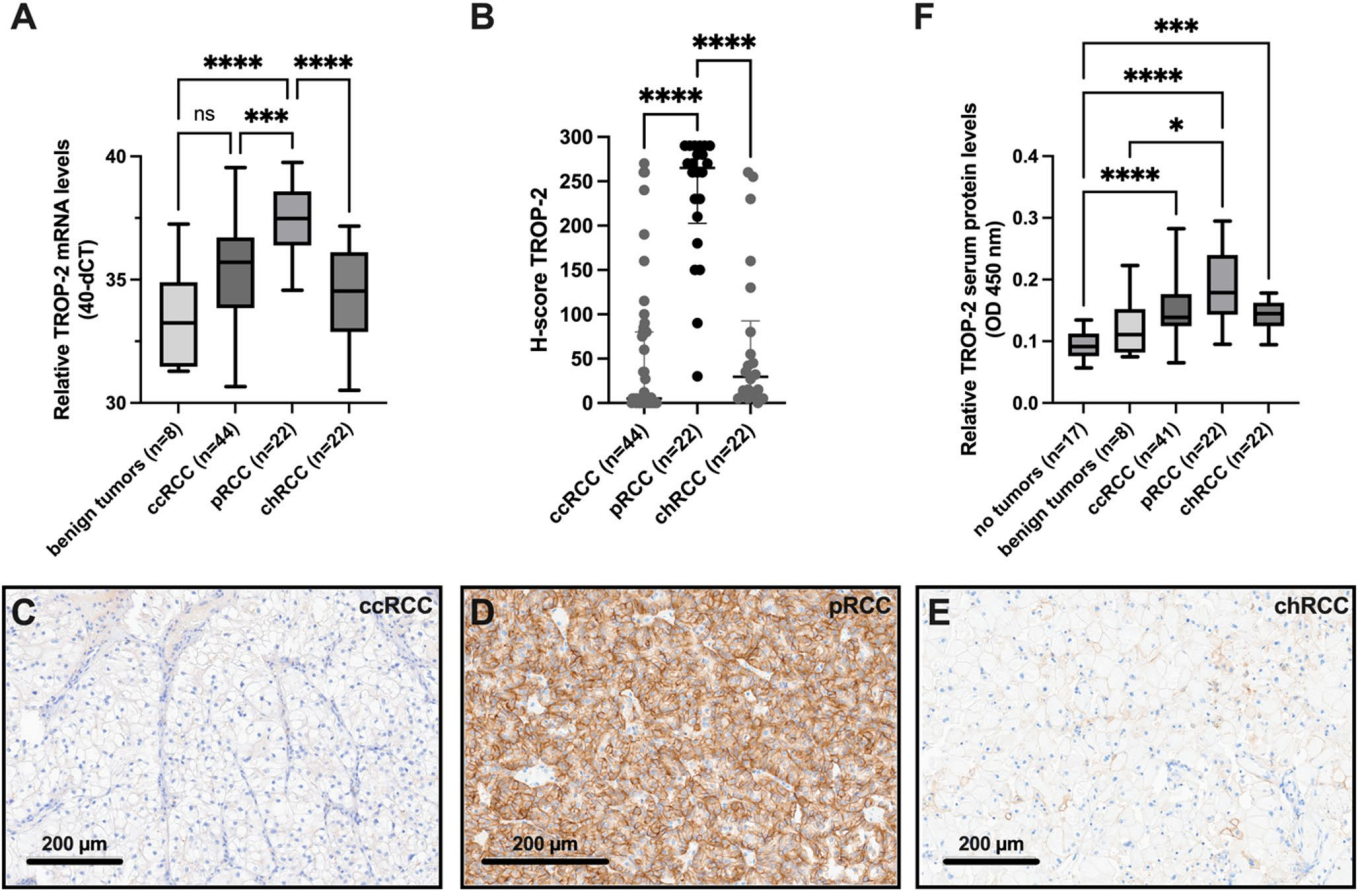
TROP-2 mRNA and protein expression in renal tumor subtypes assessed by RT-qPCR, IHC, and ELISA. **A** TROP-2 mRNA levels are significantly elevated in pRCC (*n* = 22) compared to ccRCC (*n* = 44), chRCC (*n* = 22) and benign tumors (*n* = 8). **B** Moderate to strong membranous TROP-2 expression in all pRCC cases (*n* = 20/22) [median H-score = 265; interquartile range (IQR) 202.5–290], while expression is absent or weak in ccRCC and chRCC. **C**–**E** Representative IHC images show heterogenous TROP-2 staining: (**C**) ccRCC, with a H-score of 5, (**D**) pRCC, with a H-score of 265 and (**E**) chRCC, with a H-score of 29.5 (magnification 200x). **F** Serum TROP-2 protein levels are significantly higher in pRCC patients (*n* = 22) compared to individuals without tumors (*n* = 17) and those with benign renal tumors (*n* = 8). RCC, renal cell carcinoma; pRCC, papillary RCC; ccRCC, clear-cell RCC; chRCC, chromophobe RCC; Kruskal-Wallis test: *p < 0.05, ***p < 0.001, ****p < 0.0001

TROP-2 mRNA expression was assessed across renal tumor subtypes and benign lesions using tumor tissue from 88 RCC patients and eight individuals with benign renal tumors. TROP-2 expression was significantly higher in pRCC compared to ccRCC, chRCC, and benign renal tumors (Kruskal-Wallis test, *p* < 0.001; Fig. 1A). Receiver operating characteristic (ROC) analysis demonstrated strong diagnostic potential for differentiating pRCC from benign tumors (AUC = 0.94, 95% CI 0.84-1.00, *p* = 0.0002; Fig. S1A). Similarly, TROP-2 expression distinguished pRCC from ccRCC and chRCC (AUC = 0.80 and 0.90, respectively; *p* < 0.0001 for both; Fig. S1B, C).

### Broad TROP-2 protein expression in papillary RCC

Immunohistochemical analysis revealed a distinct pattern of membranous TROP-2 protein expression across RCC subtypes. Among pRCC cases, 90.9% (20/22) exhibited moderate to strong expression (H-score ≥ 100), with a median H-score of 265 (interquartile range [IQR]: 202.5–290) (Fig. 1B). In contrast, the majority of ccRCC (81.8%, 36/44) and chRCC (77.3%, 17/22) samples showed absent or weak expression, with median H-scores of 5 (IQR: 0–80) and 29.5 (IQR: 9.25–92.5), respectively (Kruskal-Wallis test, *p* < 0.0001; Fig. 1B). Representative staining patterns are illustrated in Fig. 1C–E.

Within the pRCC subgroup, TROP-2 expression was significantly associated with local tumor burden (Chi-squared test, *p* = 0.002), but not with ISUP grade or the presence of metastases (Table S1).

### Serum TROP-2 protein levels in RCC patients

Serum TROP-2 levels were quantified in 85 RCC patients, eight individuals with benign renal tumors, and 17 tumor-free controls. RCC patients exhibited significantly higher serum TROP-2 levels compared to controls (Kruskal-Wallis test, *p* < 0.001; Fig. 1F). Levels were also elevated in pRCC patients compared to those with benign tumors (*p* < 0.05), though no significant differences were observed between pRCC and other RCC subtypes (pRCC vs. ccRCC: *p* = 0.171; pRCC vs. chRCC: *p* = 0.254).

ROC analysis supported the diagnostic utility of serum TROP-2, with an AUC of 0.96 (95% CI 0.90-1.00, *p* < 0.0001) for distinguishing pRCC from healthy controls, and an AUC of 0.83 (95% CI 0.67–0.99, *p* = 0.004) for pRCC versus benign tumors (Fig. S1D and S1E).

In the pRCC subgroup, Spearman correlation and linear regression analyses demonstrated strong positive correlations between TROP-2 mRNA levels, serum protein concentrations, and H-scores, with correlation coefficients of 0.70 (*p* = 0.0003, R²=0.58) and 0.78 (*p* = 0.0001, R²=0.52), respectively (Fig. S2).

### On-target efficacy of sacituzumab Govitecan in RCC cell lines

**Fig. 2:**
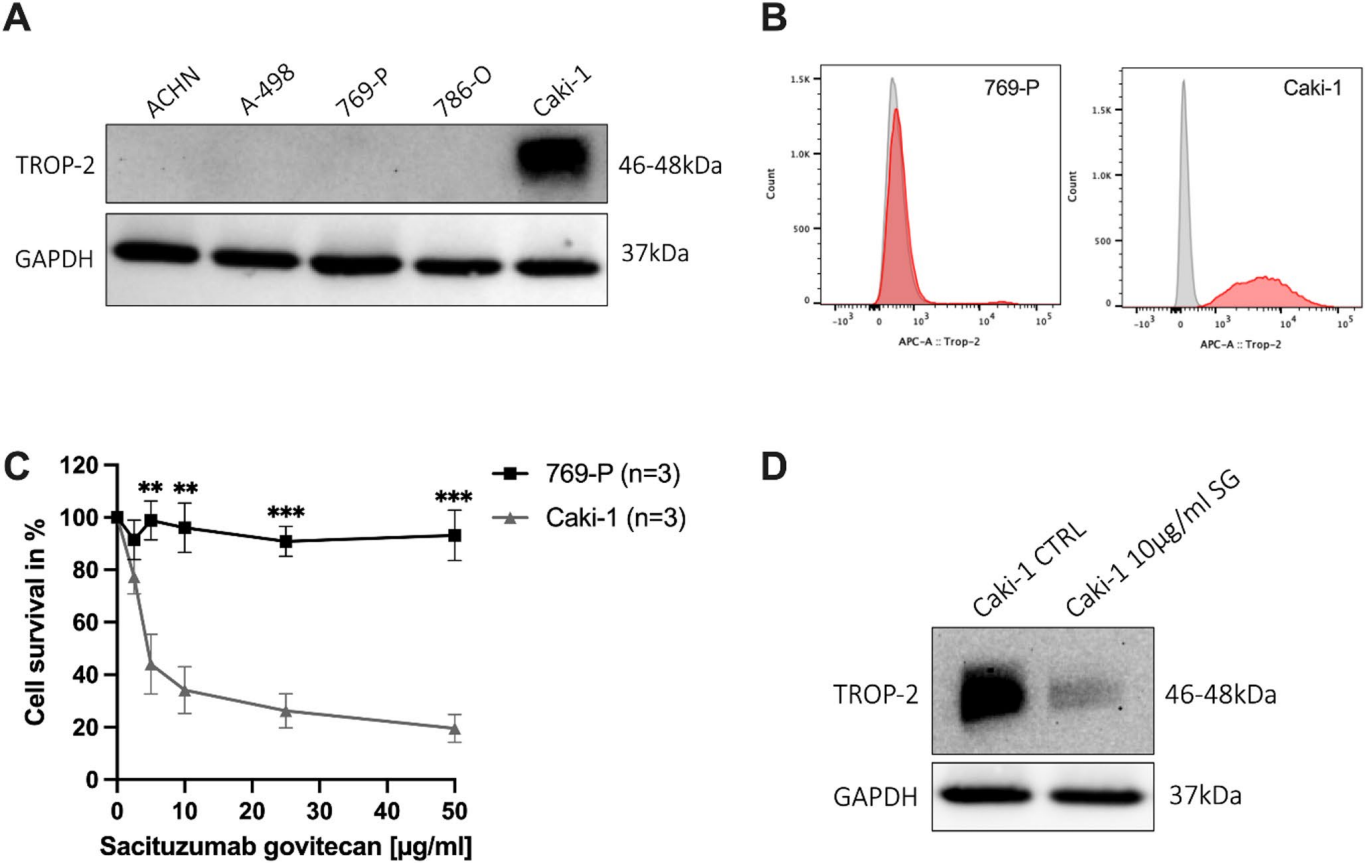
On-target efficacy of Sacituzumab govitecan in RCC cell lines. **A** TROP-2 protein expression levels in a panel of RCC cell lines (ACHN, A-498, 769-P, 786-O, and Caki-1) using Western blot, with only Caki-1 cells displaying a strong TROP-2 expression. Detection of GAPDH served as loading control. **B** Normalized histogram illustrating membranous TROP-2 expression detected by flow cytometry in 769-P and Caki-1 (plus unstained control as reference). **C** SG led to significant growth inhibition in the TROP-2-expressing Caki-1, while TROP-2 negative 769-P cells were found to be resistant to SG treatment (unpaired t-test, *p* < 0.01). **D** SG led to a reduced TROP-2 expression in the TROP-2 expressing RCC cell line Caki-1 shown using Western blot. RCC, renal cell carcinoma; SG, Sacituzumab govitecan; multiple unpaired t-tests: *p < 0.05, ***p < 0.001, ****p < 0.0001

Western blot and flow cytometric analyses were performed to assess TROP-2 protein expression in five RCC cell lines. Only Caki-1 cells, derived from a metastatic ccRCC lesion, exhibited strong TROP-2 expression (Fig. 2A and B). Other lines, including 769-P, A-498, ACHN, and 786-O, demonstrated absent expression (Fig. 2A and B).

To evaluate therapeutic efficacy, TROP-2-positive Caki-1 cells and TROP-2-negative 769-P cells were treated with increasing concentrations of SG (0–50 µg/mL) for 72 h. Cell viability was assessed via MTT assay (*n* = 3 independent experiments). SG elicited a dose-dependent reduction in Caki-1 cell viability, with a significant decrease observed at higher concentrations compared to untreated controls (unpaired t-test, *p* < 0.01; Fig. 2C). No significant cytotoxicity was observed in 769-P cells, confirming TROP-2-dependent SG activity.

Finally, Western blotting revealed a marked decrease in TROP-2 protein levels in SG-treated Caki-1 cells relative to untreated controls (Fig. 2D), consistent with target internalization and degradation, supporting an on-target mechanism of action.

## Discussion

This study provides preclinical evidence supporting the relevance of TROP-2 as a therapeutic target in pRCC, highlighting its potential role in the future landscape of targeted therapies for nccRCC. Our findings demonstrate a distinct overexpression of TROP-2 at both the mRNA and protein levels in pRCC, with limited expression in other RCC subtypes and benign renal tumors. Notably, functional analyses revealed that SG, a TROP-2-directed ADC, exhibits cytotoxic effects in TROP-2-positive RCC cell lines, indicating a potential role for ADC-based therapies in this context.

While our data provide valuable insights, several aspects warrant discussion within a broader clinical context. First, regarding current treatment strategies in pRCC, the ARON-1 trial recently demonstrated superior outcomes for IO-based combination therapies compared to TKI monotherapy in metastatic pRCC, including improved PFS and ORRs [1, 3]. These results underscore the increasing relevance of IO in nccRCC and support a paradigm shift away from TKI monotherapy as a first-line standard.

Despite these advances, there remains a substantial unmet need for effective second-line therapies and molecularly guided treatment strategies. Precision oncology in RCC has historically focused on clear cell histology, but emerging evidence suggests that pRCC harbours distinct molecular alterations, such as MET, CDKN2A, and NRF2 pathway aberrations, which may inform future treatment approaches [21]. In this context, our study contributes to ongoing efforts to identify new biomarkers and therapeutic targets that may enhance outcomes for patients with TROP-2-positive pRCC.

Our findings of TROP-2 expression across RCC subtypes aligns with previous large-scale tissue-based studies, including the work of Dum et al., which reported TROP-2 positivity in approximately 75% of pRCC cases based on immunohistochemistry [12, 22]. This further supports our observation that TROP-2 may serve as a subtype-selective marker. However, the presence of TROP-2-negative pRCC tumors, as also noted by Dum et al. [12], underscores the importance of biomarker-based patient stratification in future clinical trials.

Moreover, while the detection of soluble TROP-2 in serum is promising, we caution against interpreting it as a standalone diagnostic biomarker. The observed correlation between serum and tissue expression warrants further investigation in larger, prospective cohorts. At present, TROP-2 may better serve as a companion diagnostic to guide ADC therapy rather than a primary screening tool for RCC.

From a translational perspective, our findings provide a rational for clinical evaluation of TROP-2-targeted therapies in pRCC. Although SG is currently approved for breast and urothelial cancers [10, 11], the preclinical efficacy observed here supports its investigation in biomarker-enriched clinical trials for pRCC. However, further validation in TROP-2-high models, including patient-derived xenografts and prospective clinical cohorts, is essential before clinical implementation.

We acknowledge several limitations of our study. First, its retrospective, single-center design may introduce selection bias, potentially limiting the generalizability of the findings. Second, our functional analyses were based on RCC cell lines with diverse and potentially unrepresentative origins. While ACHN is derived from a pleural metastasis of papillary RCC, Caki-1 originates from a cutaneous metastasis of clear cell RCC, which may not accurately reflect the biology of the corresponding primary tumor. Given that TROP-2 overexpression has been documented in squamous cell carcinomas, including penile cancer, it is conceivable that the cutaneous localization of the Caki-1 metastasis contributed to its TROP-2 positivity. While this observation does not diminish the rationale for TROP-2 targeting in RCC, it highlights the necessity for validation in primary pRCC-derived models.

In conclusion, the strong TROP-2 expression in pRCC, together with its demonstrated functional relevance in vitro, provides compelling preclinical evidence supporting its potential as a therapeutic target for ADC therapy.

## Supplementary Information

Below is the link to the electronic supplementary material.

Supplementary Material 1

## Data Availability

The data that support the findings of this study are available from the corresponding author upon reasonable request.

## Abbreviations

ADC: Antibody-drug-conjugate
ccRCC: Clear cell renal cell carcinoma
chRCC: Chromophobe renal cell carcinoma
ELISA: Enzyme-linked immunoassay
FFPE: Formalin-fixed paraffin-embedded
IHC: Immunohistochemistry
IO: Immune-oncology
nccRCC: Non-clear cell renal cell carcinoma
OS: Overall survival
RCC: Renal cell carcinoma
RT-qPCR: Reverse transcription quantitative polymerase chain reaction
PFS: Progression-free survival
pRCC: Papillary renal cell carcinoma
SG: Sacituzumab govitecan
TKI: Tyrosine kinase inhibitors
TNBC: Triple-negative breast cancer
TROP-2: Trophoblast cell surface antigen-2
UC: Urothelial cancer
