# Supplementary material for: TROP-2 overexpression in papillary renal cell carcinoma supports its potential as a therapeutic target for antibody-drug-conjugate therapy
Source: World J Urol. 2025 Sep 1;43(1):522. doi: 10.1007/s00345-025-05880-2 (PMC12401752; doi:10.1007/s00345-025-05880-2)
Supplement: Supplementary file 1 — Supplementary Material 1 [file 345_2025_5880_MOESM1_ESM.docx]

**Table S1**. Clinicopathological characteristics for pRCC patients in dependence of TROP-2 expression.

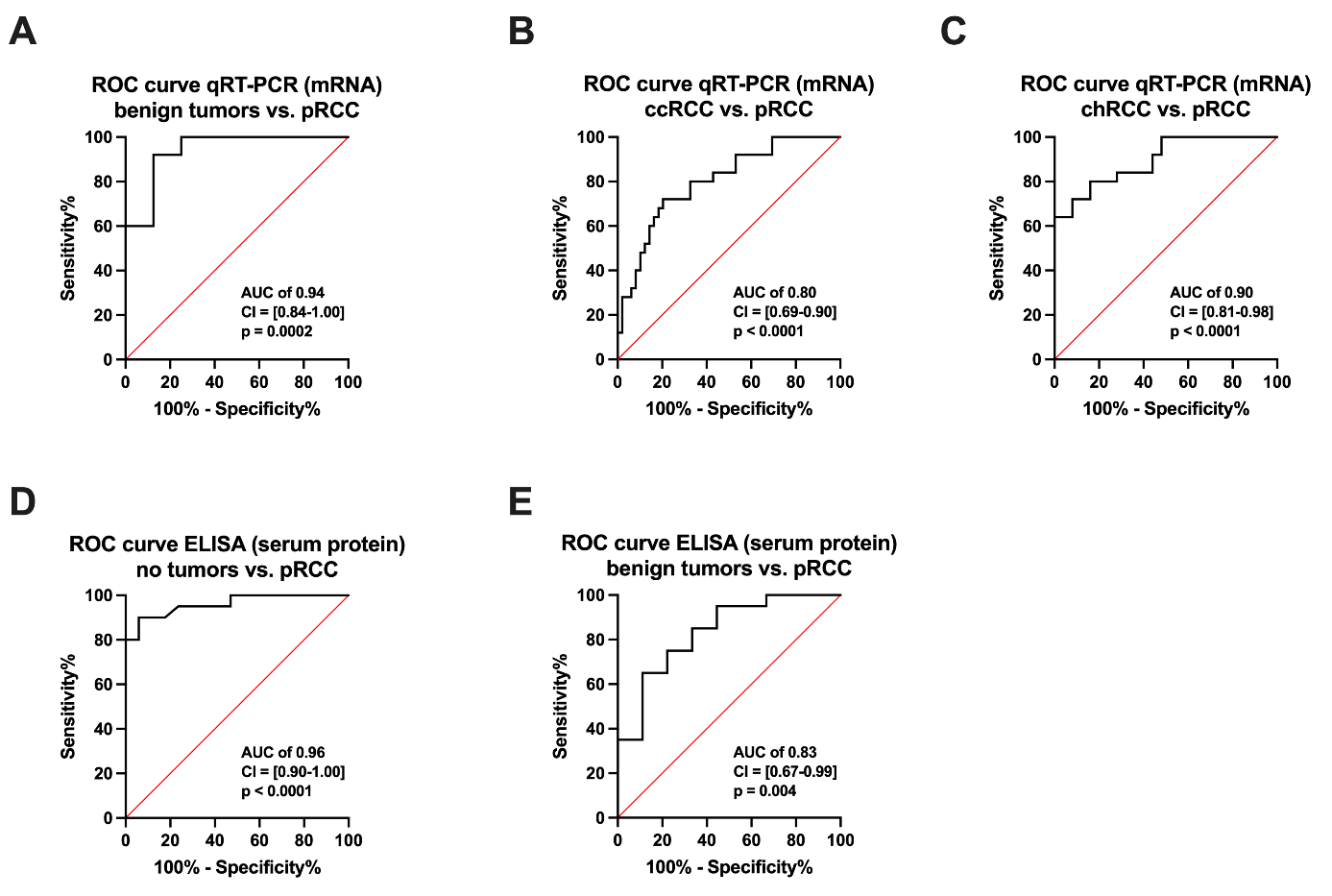

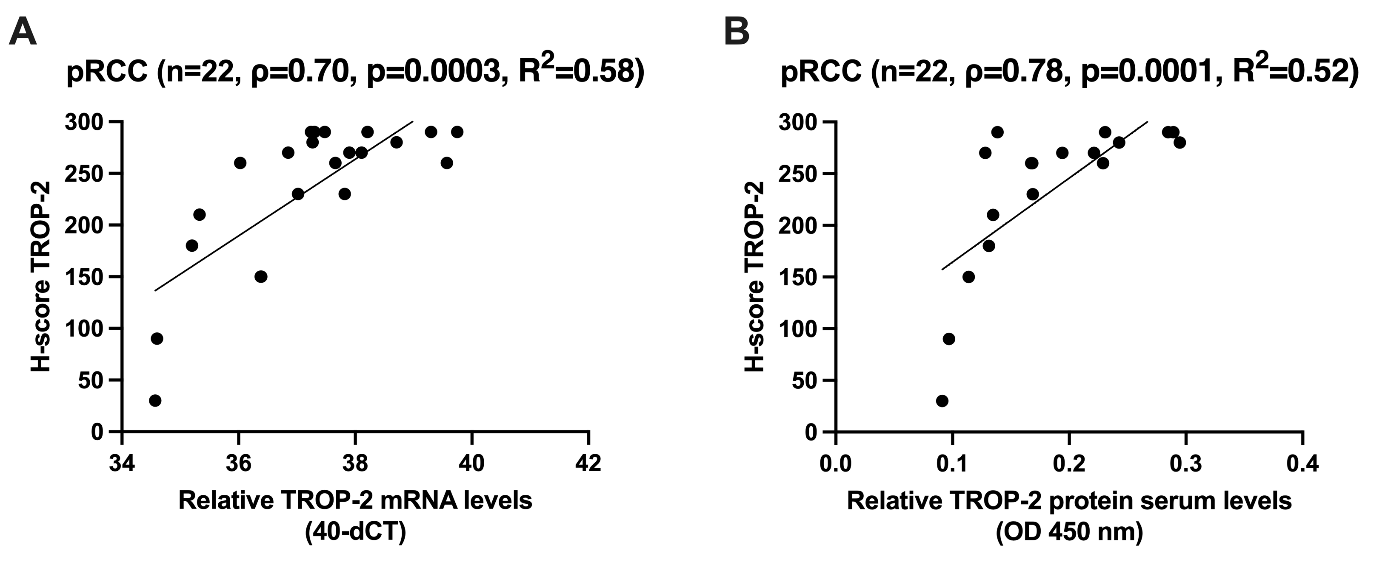
**Figure S1. ROC curve analyses demonstrating the strong diagnostic potential of TROP-2.**

**Figure S2. Spearman correlation and linear regression analyses between TROP-2 mRNA, serum protein levels, and the H-score.** Strong positive correlations between TROP-2 mRNA levels and membranous expression, as well as between TROP-2 serum protein levels and H-score, with Spearman coefficients of 0.70 (p=0.0003, R^2^=0.58), and 0.78 (p=0.0001, R^2^=0.52).
